# Specific Inhibition of the Redox Activity of Ape1/Ref-1 by E3330 Blocks Tnf-Α-Induced Activation of Il-8 Production in Liver Cancer Cell Lines

**DOI:** 10.1371/journal.pone.0070909

**Published:** 2013-08-15

**Authors:** Laura Cesaratto, Erika Codarin, Carlo Vascotto, Antonio Leonardi, Mark R. Kelley, Claudio Tiribelli, Gianluca Tell

**Affiliations:** 1 Dipartimento di Scienze Mediche e Biologiche, Università di Udine, Udine, Italy; 2 Fondazione Italiana Fegato, AREA Science Park, Basovizza, Trieste, Italy; 3 Dipartimento di Patologia Cellulare e Molecolare ‘L. Califano’ Università “Federico II” di Napoli, Napoli, Italy; 4 Department of Pediatrics, Herman B Wells Center for Pediatric Research, Indiana University, Indianapolis, Indiana, United States of America; 5 Dipartimento Scienze Cliniche, Università di Trieste, Trieste, Italy; Institut national de la santé et de la recherche médicale – Institut Cochin, France

## Abstract

APE1/Ref-1 is a main regulator of cellular response to oxidative stress *via* DNA-repair function and co-activating activity on the NF-κB transcription factor. APE1 is central in controlling the oxidative stress-based inflammatory processes through modulation of cytokines expression and its overexpression is responsible for the onset of chemoresistance in different tumors including hepatic cancer. We examined the functional role of APE1 overexpression during hepatic cell damage related to fatty acid accumulation and the role of the redox function of APE1 in the inflammatory process. HepG2 cells were stably transfected with functional and non-functional APE1 encoding plasmids and the protective effect of APE1 overexpression toward genotoxic compounds or FAs accumulation, was tested. JHH6 cells were stimulated with TNF-α in the presence or absence of E3330, an APE1 redox inhibitor. IL-8 promoter activity was assessed by a luciferase reporter assay, gene expression by Real-Time PCR and cytokines (IL-6, IL-8, IL-12) levels measured by ELISA. APE1 over-expression did not prevent cytotoxicity induced by lipid accumulation. E3330 treatment prevented the functional activation of NF-κB *via* the alteration of APE1 subcellular trafficking and reduced IL-6 and IL-8 expression induced by TNF-α and FAs accumulation through blockage of the redox-mediated activation of NF-κB. APE1 overexpression observed in hepatic cancer cells may reflect an adaptive response to cell damage and may be responsible for further cell resistance to chemotherapy and for the onset of inflammatory response. The efficacy of the inhibition of APE1 redox activity in blocking TNF-α and FAs induced inflammatory response opens new perspectives for treatment of inflammatory-based liver diseases.

## Introduction

Non-alcoholic steatohepatitis (NASH) defines a distinct hepatic disorder observed in patients without a history of alcohol abuse that histologically resembles alcohol-induced liver damage and includes cellular damage, inflammation and fibrosis [1] and may evolve towards cirrhosis, liver failure and HCC [2]. The mechanisms of this progression and the pathogenesis of NASH are still poorly understood although oxidative stress, generated as a consequence of mitochondrial impairment, seems to be directly linked with the onset of the inflammatory circuits responsible for the progression of this pathology. One of the key pro-inflammatory cytokines that appears to be involved in modulating the inflammatory response in several forms of liver injury is interleukin-8 (IL-8) [3], a CXC chemokine, that recruits and activates neutrophils, basophils and T cells [4]. Since patients with NASH have significantly elevated serum levels of IL-8 compared with healthy individuals, IL-8 may play a key role in the pathogenesis of NASH [5]. In different hepatic *in vitro* models, lipid accumulation can stimulate IL-8 production [6] through activation of NF-κB [7]. In the rat liver, free Fatty Acids (FAs) activate the NF-κB pathway and increase the expression of some pro-inflammatory cytokines (TNF-α, IL-1β, IL-6) [8], [9].

The Apurinic apyrimidinic Endonuclease/Redox effector factor 1 (APE1/Ref-1) is a multifunction protein that acts as a master regulator of cellular response to oxidative stress conditions and contributes to the maintenance of genome stability. APE1 is involved in both the base excision repair (BER) pathways of DNA lesions, acting as the major apurinic/apyrimidinic (AP) endonuclease, and in transcriptional regulation of gene expression as a redox co-activator of different transcription factors, such NF-κB and others [10], [11]. In gastric epithelial cells APE1 plays a leading role in controlling the onset of oxidative stress-based inflammatory processes through modulating NF-κB-mediated IL-8 gene expression [12]. APE1 expression is also up-regulated during hepatic lipid accumulation in NASH patients [13], although it is still unknown whether this upregulation has a causal role in the onset of NASH or is associated to a protective function on lipid accumulation cytotoxic effect.

APE1 is upregulated in liver cancers [14], but the functional role of this overexpression in tumor pathogenesis and progression is not yet clear. APE1 redox function is exerted through a novel redox-based mechanism involving three cysteine residues (i.e. C65, C93 and C99) [15]. Recent *in vitro* studies demonstrated that APE1 adopts different unfolded conformations depending on the redox state of its Cys residues [15]. The (E)-3-(2-[5,6-dimethoxy-3-methyl-1,4-benzoquinonyl])-2-nonyl propenoic acid (E3330) has been reported to directly bind APE1 protein and to inhibit its redox activity, without interfering with its endonuclease activity, by increasing the formation of disulfide bonds involving the redox-active Cys65, altering the folding of APE1 protein and decreasing the protein redox active population [16], thus impacting on APE1 subcellular trafficking [17]. E3330 holds clinical therapeutic potential as a specific inhibitor of APE1 redox function [18]. The importance of this function is highlighted by results demonstrating that NF-κB-mediated gene expression is regulated by APE1 redox activity, without effects on IκBα degradation [19]. E3330 was also found to selectively inhibit growth/migration of human pancreatic cancer cells [20], suggesting that the APE1 redox function could represent a good candidate for inhibition of tumor invasion and metastasis. E3330 suppressed inflammatory response in activated macrophages [21], suggesting the possible use of E3330 to reduce the inflammatory processes in liver diseases, such as those associated to NASH.

In this study, we used *in vitro* models of fat overloading obtained by exposing hepatic cells to a mixture of long-chain FAs (palmitic (C16∶0) and oleic (C18∶1) acids that are the most abundant FAs in liver triglycerides [22], [23]. Additionally, the present study was aimed at examining the functional role of APE1 overexpression during hepatic cell damage related to lipid accumulation and the role of the redox function of APE1 in the inflammatory process triggered by TNF-α and FAs accumulation. Our data demonstrate that APE1 overexpression does not protect from FAs induced cell damage and that APE1 and NF-κB play an essential role in TNF-α-induced transcriptional activation of IL-8 gene expression in hepatic cancer cell lines. Inhibition of APE1 redox activity by the redox inhibitor E3330 is efficient in preventing both TNF-α or lipid accumulation induced activation of IL-8 expression at the transcriptional level through the blockage of the redox-mediated activation of NF-κB.

## Materials and Methods

### Cell culture and treatments

In this study, we used the Huh-7 (differentiated hepatocyte derived cellular carcinoma cell line) [24], the HepG2 (differentiated hepatocellular carcinoma) [25] and the JHH6 (undifferentiated hepatocellular carcinoma) [26] as models of the liver tumorigenic process. Huh-7 and JHH6 were purchased from Health Science Research Resources Bank (Osaka, Japan) while HepG2 from ATCC. Huh-7 and HepG2 cells were cultured in Dulbecco's modified Eagle's medium (EuroClone, Pero, IT), JHH6 cells were cultured in William's medium E (Sigma-Aldrich, St Louis, MO), both supplemented with 10% fetal bovine serum, 2 mM L-glutamine, 100 U/ml penicillin and 100 µg/ml streptomycin (Euroclone, Milan, IT).

HepG2 cell clones were cultured at a density of 70000 cells/cm^2^ and were treated with different doses of methyl methanesulfonate (MMS) or 2.5 mM H_2_O_2_ (both reagents are distributed by Sigma-Aldrich) for 2 h or 1 h respectively.

For etoposide treatment, HepG2 cell clones were trypsinized and 400000 cells were seeded on glass coverslips in 6-wells culture plates. After 24 h, medium was replaced by Dulbecco's modified Eagle's medium (DMEM) with or without etoposide (Sigma, St Louis, MO) 50 µM for 1 h. (2E)-3-[5-(2,3-dimethoxy-6-methyl 1,4-benzoquinoyl)] -2-nonyl-2-propenoic acid (E3330; custom synthesized) [16] was solubilized in DMSO. Treatment with E3330 was performed in serum-free William's medium E in order to prevent the serum albumin levels to affect E3330 final concentration.

Treatment with recombinant human TNF-α (Peprotech Inc., Rocky Hill, NJ) was performed at 2000 U/ml in serum-free William's medium E to minimize serum-induced IL-8 release.

To induce fat-overloading of cells, JHH6 or HepG2 cell clones were seeded at a density of 14000 and 57000 cells/cm^2^ respectively and after 24 h cells were exposed to a mixture of long-chain FAs (oleate and palmitate) at 2∶1 ratio. Stock solutions of 100 mM oleic acid (Sigma-Aldrich) and 100 mM palmitic acid (Sigma-Aldrich), prepared in DMSO, were conveniently diluted in William's medium E or DMEM high glucose containing 60 µM albumin from bovine serum (Sigma-Aldrich) to obtain the desired final concentrations. With mixtures of FAs and albumin, the uptake is a function of free fatty acid (FAs), which is the monomeric form in equilibrium with albumin-bound FAs [27].

### Generation of APE1 overexpressing hepatic cell lines

For generation of APE1 overexpressing cell lines, an APE1 expression vector was generated by cloning an EcoRI-BamHI fragment from pFLAG-CMV-5.1/APE1 (Sigma, Milan, IT) into p3XFLAG-CMV-14 vector (Sigma). The APE1^NΔ33^ deletion mutant was generated by PCR and subcloning from the full-length cDNA sequence. Correctness of the cloning procedure was confirmed by DNA sequencing. Then, HepG2 cells were transfected with p3XFLAG-CMV/APE1, the wild-type APE1 (APE1^WT^) and the deletion mutant (APE1^NΔ33^), previously digested with ScaI (Fermentas, St. Leon Rot, UK); 48 h after transfection, the cells were subjected to selection with G418 (Invitrogen, Milan, Italy) for 14 days and selected for the acquired resistance. Individual clones were isolated by using cell cloning cylinders (Sigma), transferred and grown stepwise into 24-well, 12-well, and 6-well plates for expansion to 10^7^ cells in the presence of selective antibiotic. As a control, we used cell clones transfected with the p3XFLAG-CMV-14 empty vector. Geneticin (G418) (PAA Laboratories GmbH, Pasching, AT) was added to the cell culture medium at the final concentration of 300 µg/ml during cell growth. Total cellular extracts were analysed for APE1 expression by immunoblotting, thus revealing the expression of the ectopic flagged WT and the mutant form of the protein.

### MTT and cell growth assays

To determine the viability of HepG2 cells over-expressing APE1 protein, a 3(4,5-dimethylthiazolyl-2)-2,5 diphenyl tetrazolium (MTT) assay was performed [28]. HepG2 control and over-expressing APE1 clones were seeded onto 96 multi-well plates at a density of 70000 cells/cm^2^ for each well. The day after, cells were incubated with MMS or H_2_O_2_ as indicated. After treatments, in each well 1/10 volume of MTT solution (4 mg/ml in PBS) was added and incubated for 2 h at 37°C. Then, the supernatant was removed and an equal volume of DMSO was added to the cells and the MTT formazan was dissolved by pipetting. The absorbance was measured on an ELISA plate reader (EL808 Ultra Microplate Reader Bio-tek Instruments, Winooski, VT) with a test and reference wavelength of 570 and 630 nm, respectively.

For trypan blue exclusion experiments, cells were trypsinized, resuspended in 0.08% w/v trypan blue (Sigma) in complete medium, and counted after 3–5 min of incubation.

Data were expressed as percentage of surviving cells compared with the untreated control.

### MTS assay

The day before treatment, JHH6 cells were seeded at a density of 26000 cells/cm^2^. To evaluate the effect of treatment in terms of cell viability, the CellTiter 96^®^ AQ_ueous_ One Solution Cell Proliferation Assay (Promega Corporation, Madison, WI) was used according to the manufacturer's instructions. This assay contains a novel tetrazolium compound [3-(4,5-dimethylthiazol-2-yl)-5-(3-carboxymethoxyphenyl)-2-(4-sulfophenyl)-2H-tetrazolium, inner salt; MTS] that is bioreduced by cells into a colored formazan product that is soluble in cell culture medium and can be quantified by reading the absorbance at 490 nm.

### Nile Red staining

The lipid content in cultured cells was determined fluorometrically by using Nile Red staining (Sigma-Aldrich, Milan, IT), a vital lipophilic and selective fluorescent stain for intracellular lipid droplets accumulation [29].

Stock solutions of Nile Red (100 or 1000 µg/ml) in acetone were prepared and stored protected from light. Staining has been carried out on fixed cells (1.5% glutaraldehyde, 5 min). Cell monolayers were washed twice with PBS, treated for 5 min with 0,1% Triton X-100 in PBS and incubated for 1 h with Nile Red solution to effect a 1∶100 dilution in PBS. After Nile Red treatment, nuclei were stained by 5 min incubation in 300 nM solution of 4′, 6′-diamidino-2-phenylindole dihydrochloride (DAPI) (Sigma) in PBS. Monolayers were then washed three times in PBS and used for fluorescence microscopy. Immunofluorescent images were collected using a confocal microscope (Leica DM IRB/E, Wetzlar, Germany) at the excitation wavelength, 450–500 nm and emission wavelength >528 nm.

### Preparation of total cell extracts

For preparation of total cell lysates, cells were harvested by trypsinization and centrifuged at 250×*g* for 5 min at 4°C. Supernatant was removed, and the pellet was washed once with ice-cold phosphate-buffered saline (PBS) and then centrifuged again as described before. Cell pellet was resuspended in lysis buffer containing 50 mM Tris-HCl (pH 7.4), 150 mM NaCl, 1 mM EDTA, and 1% (wt/vol) Triton X-100 supplemented with 1x protease inhibitor cocktail (Sigma), 0.5 mM phenylmethylsulfonyl fluoride (PMSF), 1 mM NaF and 1 mM Na_3_VO_4_, at a cell density of 10^7^ cells/ml for 30 min at 4°C. After centrifugation at 12,000×*g* for 30 min at 4°C, the supernatant was collected as total cell lysate. The protein concentration was determined using Bio-Rad protein assay reagent (Bio-Rad, Hercules, CA).

### Western blot analysis

The indicated amounts of cell extracts were electrophoresed onto a 12% SDS-PAGE. Proteins were then transferred to nitrocellulose membranes (Schleicher & Schuell, Keene, NH). Membranes were saturated by incubation at 4°C overnight with 5% (wt/vol) nonfat dry milk in PBS–0.1% (wt/vol) Tween 20 and then incubated with the polyclonal anti-APE1 antibody [17] for 3 h. After three washes with PBS–0.1% (wt/vol) Tween 20, membranes were incubated with an anti-rabbit Ig coupled to peroxidase (Sigma). Upon 60 min of incubation at room temperature, the membranes were washed three times with PBS–0.1% Tween 20 and the blots were then developed using the ECL enhanced chemiluminescence procedure (PIERCE, Rockford, IL). Normalization was performed with the polyclonal anti-β-tubulin antibody (Sigma). Blots were quantified by using a Chemi DOC XRS densitometer (Bio-Rad).

### Immunofluorescence and confocal analysis

Twenty-four hours before the experiment, cells seeded at 4×10^5^ cells/cm^2^, were grown on glass cover slips. For APE1 immunofluorescence experiments, HepG2 cell clones were fixed in 4% (wt/vol) paraformaldehyde for 20 min at room temperature, permeabilized for 5 min with PBS-0.25% (wt/vol) Triton X-100 and then incubated for 30 min with 10% (v/v) fetal bovine serum (FBS) in PBS (blocking solution) to block unspecific binding of the antibodies. Cells were then incubated overnight at 4°C with the anti-FLAG^®^ M2 Monoclonal Antibody-FITC Conjugate (Sigma) diluted 1∶100 in blocking solution. After washing, a second blocking step for 30 min in the dark was performed, and then the cells were incubated for 3 h with the second primary antibody, rabbit-polyclonal to Histone H3 (Acetyl K18) (Abcam, Cambridge, UK) in blocking solution. After washing, cells were incubated for 90 min with secondary antibody Alexa Fluor 546-conjugated goat anti-rabbit (1∶500; Molecular Probes, Monza, IT). The preparations were then washed with PBS three times for 5 min each in the dark. Nuclei were counterstained by 5 min of incubation in 300 nM solution of 4′, 6′-diamidino-2-phenylindole dihydrochloride (DAPI) (Sigma) in PBS. The preparations were then washed three times in PBS for 5 min. The microscope slides were then mounted onto slides with an anti-fade reagent. Coverslips were visualized through a Leica TCS SP laser-scanning confocal microscope (Leica Microsystems, Wetzlar, Germany) equipped with a 488-nm argon laser, a 543-nm HeNe laser, and a 63x oil fluorescence objective.

For γ-H2A.X immunofluorescence experiments, control and etoposide-treated cells were washed with PBS, fixed for 20 min with 4% (wt/vol) paraformaldehyde in PBS, and permeabilized for 5 min with 0.25% (wt/vol) Triton X-100 in PBS. Slides were blocked with 10% (v/v) FBS in PBS, at 4°C, overnight, incubated for 2 h at room temperature with anti-γ-H2A.X antibody (Stressgen, Ann Harbor, MI) and washed with 0.1% (wt/vol) Triton X-100 in PBS. Anti-γ-H2A.X antibody were used at 1∶500 dilution. For detection, cells were incubated with Alexa Fluor-488-labelled anti-mouse secondary antibody (Invitrogen, Monza, IT). Nuclei were counterstained incubating cells with 0.3 µg/ml propidium iodide (PI) for 5 min, at 37°C. Cells were washed and mounted in Mowiol^®^ 4–88 (Sigma) supplemented with 1∶5 DABCO (Sigma) as anti-fade reagent. Images were collected using a confocal microscope.

Quantification of γ-H2A.X foci was carried out by BD Pathway 855, using 20X objective. Automated image analysis was performed with customizable and highly flexible software tools. Nuclear boundaries were generated using the Hoechst images. The γ-H2A.X images were acquired and data intensity was analyzed using BD Pathway™ system software within the nuclear boundaries.

### Transient transfection experiments

The constructs of human IL-8 promoter, −1498/+44 hIL-8/Luc and −162/+44 hIL-8 ΔNF-κB/Luc, were kindly provided by Dr. S.E. Crowe [30].

One day before transfection, JHH6 cells were seeded in triplicate in 96-well plates at a density of 31000 cells/cm^2^. Then cells were transiently transfected with 200 ng of total DNA (hIL-8/Luc promoter and pRL-CMV Renilla luciferase constructs in a ratio of 49∶1) per well, using Lipofectamine 2000 reagent (Invitrogen, Carlsbad, CA) according to the manufacturer's instructions. The transfection reagent was removed 4 h post-transfection and cells were incubated with complete medium for 16 h. The following day, cells were washed twice with PBS, pre-treated with E3330 in serum-free William's medium E and then treated with TNF-α as reported in the text. Finally cells were lysed with Dual-Glo^®^Luciferase Assay System (Promega) according to the manufacturer's instructions. The luminescence signals were quantified using a Modulus^TM^ II Microplate Multimode Reader (Turner Biosystems Inc., Sunnyvale, CA). Firefly luciferase activity was normalized to the Renilla luciferase activity.

### Real time PCR

Total RNA from cells was extracted using SV Total RNA Isolation System (Promega) according to the manufacturer's instructions. Single-stranded cDNA was obtained using the iScript^TM^ cDNA Synthesis kit (Bio-Rad Laboratories, Hercules, CA) according to the manufacturer's instructions.

Real Time quantitative PCR was performed with an CFX96^TM^ Real-Time PCR Detection Systems (Bio-Rad Laboratories); Primers used were: IL-8 For 5′-CTGGCCGTGGCTCTCTTG-3′, IL-8 Rev 5′-CCTTGGCAAAACTGCACCTT-3′; 18S For 5′-CTGCCCTATCAACTTTCGATGGTAG-3′, 18S Rev 5′-CCGTTTCTCAGGCTCCCTCTC-3′; GAPDH For 5′-CCCTTCATTGACCTCAACTACATG-3′, GAPDH Rev 5′-TGGGATTTCCATTGATGACAAGC-3′; HPRT For 5′-AGACTTTGCTTTCCTTGGTCAGG-3′, HPRT Rev 5′-GTCTGGCTTATATCCAACACTTCG-3′.

cDNA was amplified in 96-well plates using primers for IL-8, 18S, GAPDH and HPRT in separate wells using the 2X iQ^TM^ SYBR^®^ Green Supermix (Bio-Rad Laboratories) [100 mM KCl; 40 mM Tris–HCl, pH 8.4; 0.4 mM of each dNTP; 50 U/ml iTaq DNA polymerase; 6 mM MgCl_2_, SYBR Green I, 20 nM fluorescein, and stabilizers] and 300 nM specific sense and anti-sense primers in a final volume of 15 µl for each well. Each sample was analysed in triplicate. A sample without template, as negative control, and a sample with not retro-transcribed mRNA instead of template cDNA, as control for genomic DNA contamination, were included. The cycling parameters were: denaturation at 95°C for 10 s and annealing/extension at 60°C for 30 s (repeated 40 times). In order to verify the specificity of the amplification, a melt-curve analysis was performed, immediately after the amplification protocol.

### Soluble cytokines determination

IL-8 and IL-12 protein levels in TNF-α-treated cell-culture supernatants were quantified using a FlowCytomix assay kit (Bender MedSystems, Atlanta, GA), according to the manufacturer's protocol.

### Statistical analysis

Statistical analysis on biological data was performed using the Microsoft Excel data analysis program for Student's *t*-test analysis. P<0.05 or P<0.01 were considered as statistically significant.

## Results

### Expression of ectopic APE1^WT^ protein confers hepatic cells protection toward genotoxic damage

**Figure 1:**
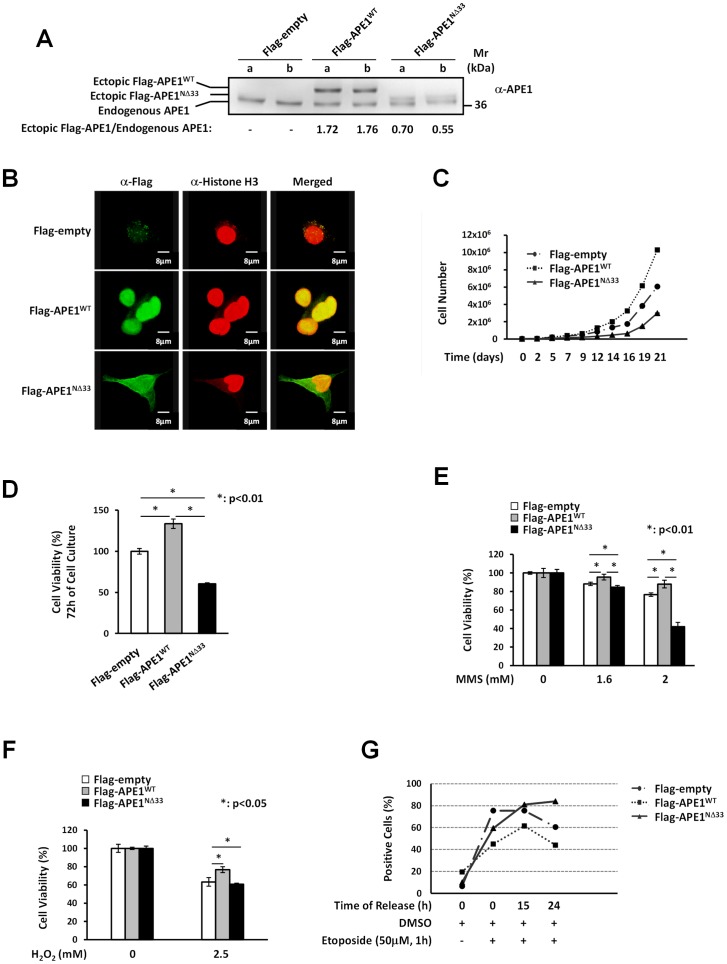
Expression of ectopic APE1^WT^ protein confers cells protection to genotoxic damage but not to FAs-induced cytotoxicity. **Panel A: ***Western Blot analysis of total cell extracts from HepG2 stable cell clones.***** Stably transfected clones have been obtained as described in Materials and Methods section. Twelve micrograms of protein extracts were separated by 12% SDS-PAGE and then transferred onto a NC membrane. The membrane was immunoblotted with anti-APE1 antibody. The values reported above refer to the ratios of the band intensities between ectopically-expressed and endogenous APE1, as measured by densitometry. The ectopic Flag-tagged recombinant protein both in the APE1^WT^ and the APE1^NΔ33^ cell clones is expressed to a similar extent at different days of cell culture. a: clones after the sixth *in vitro* passage; b: clones after the tenth *in vitro* passage. **Panel B:** ***APE1 localization within HepG2 cell clones.*** HepG2 cell clones were fixed and immunostained for Histone H3 (red) and for Flag-tagged APE1 with an α-Flag antibody (green). Merged images (yellow) show the localization of APE1^WT^ within cell nuclei and colocalization with Histone H3. The APE1^NΔ33^ deletion mutant colocalizes with Histone H3 within cell nuclei but show also cytoplasmic positivity. **Panel C:** ***Growth curve of HepG2 cell clones.*** HepG2 empty clone, APE1^WT^ clone and APE1^NΔ33^ clone cells were seeded into each well of a 24-well plate and cell growth was monitored every two or three days as indicated, by trypan blue exclusion. APE1^NΔ33^ cells (triangle) grew more slowly than the APE1^WT^ (square) and the empty clones (dot). **Panel D:** ***Cell growth by MTT colorimetric assay.*** Thirty thousand cells of the control (empty clone), APE1^WT^ and APE1^NΔ33^ clones were seeded in quadruplicate wells in a 96-well microculture plate. Cell viability was measured after 72 h of culture. MTT assay also revealed that APE1^NΔ33^ cell clone has a lower level of proliferation than empty and APE1^WT^ clones. Data, expressed as the percentage of cell viability with respect to the control empty clone, are the means ± SD of three independent experiments. **Panel E** ***: Effect of MMS on viability of HepG2 cell clones.*** HepG2 cell clones were treated for 2 h with 1.6 or 2.0 mM MMS and cell viability was estimated by the MTT colorimetric assay. When cells were treated with 2.0 mM MMS, cell viability was significantly decreased in the APE1^NΔ33^ cell clone but not in the APE1^WT^ clone, suggesting that the ectopic expression of APE1^WT^ protects cells against MMS-induced citotoxicity. Data shown are the means ± SD of three independent experiments. **Panel F:** ***Effect of H_2_O_2_ on viability of HepG2 cell clones***. HepG2 cell clones were treated with 2.5 mM hydrogen peroxide for 1 h, then cell viability was determined by the MTT assay. After exposure to 2.5 mM H_2_O_2_ no significant decrease in cell viability was detected for APE1^WT^ clone compared to empty and APE1^NΔ33^ cell clones. The histograms show the means ± SD of three independent experiments. **Panel G:** ***Quantification of γH2A.X foci in response to etoposide treatment.*** The γH2A.X foci were detected using immunohistochemistry and quantified by image analysis. Cells were treated with etoposide (50 µM) for 1 h and the number of double strand DNA breaks (DSBs) was determined at different times of release (0 h, 15 h and 24 h). γH2A.X foci levels remain significantly higher than controls at 15 h and 24 h in etoposide treated APE1^NΔ33^ cell clone (triangle). DNA damage was weaker for APE1^WT^ clone (square) than empty (dot) and APE1^NΔ33^ cell clones, suggesting a protective role of APE1 overexpression in DNA repair.

We first investigated the biological effects of APE1 over-expression on hepatic cells by using HepG2 cells, stably over-expressing the ectopic form of the wild type protein (APE1^WT^) or its deletion mutant (APE1^NΔ33^) lacking the first 33 residues [31]. The cell clones, in which the endogenous APE1 protein is co-expressed with an ectopic Flag-tagged recombinant APE1 protein, represent an overexpression model to test the role of functional and non-functional APE1 proteins in lipid-induced cytotoxic effect and mimics the condition found in advanced stages of liver cancer progression (Fig. 1A) [14]. We characterized both cell models for APE1 localization by immunofluorescence analysis, demonstrating that, similarly to the endogenous protein, the ectopic APE1^WT^ localized mainly within the nuclear compartment of HepG2 cell clones, while the APE1^NΔ33^ mutant showed a pan-cellular distribution in both cytoplasmic and nuclear compartments, as a consequence of the lack of the bi-partite NLS sequence [32] and as already observed in other cell systems (Fig. 1B) [33].

Then, we evaluated the effect of the over-expression of APE1^WT^ and APE1^NΔ33^ functional mutant on cell viability. Over-expression of APE1^WT^ caused an increased cell proliferation rate, while expression of the APE1^NΔ33^ protein form exhibited an apparent impairment of cell growth as compared to the control cell clone (Fig. 1C). Control cells showed an intermediate phenotype due to the expression of only the endogenous APE1 protein. Cell viability assays, evaluated through MTT analysis, showed that the impaired proliferation observed in the APE1^NΔ33^ mutant was associated to a reduced cell viability with respect to the APE1^WT^ expressing clone (Fig. 1D). These data support the conclusion that the NΔ33 deletion mutant may act as a dominant negative form of APE1 directly impacting on cell viability, as also seen in other cancer cell models [17], [34].

We then tested the ability of APE1 overexpression to protect cells from genotoxic treatments, as described for other cell models [35], [36], [37] but never in hepatic cells. To this aim, APE1^WT^ and APE1^NΔ33^ cell clones were treated with the DNA alkylating agent methyl methanesulfonate (MMS) [38], with hydrogen peroxide (H_2_O_2_) or with etoposide, which induces double strand breaks (DSBs) in DNA. Cell clones were incubated with increasing doses of MMS for 2 h, and cell viability was measured by MTT assay. Upon treatment with 2.0 mM MMS, cell viability was significantly reduced in the APE1^NΔ33^ expressing clone as compared with APE1^WT^ and control clones (Fig. 1E). Similar results were obtained in the case of H_2_O_2_ as genotoxicant (Fig. 1F). These results suggest that stably over-expressed APE1^WT^ protects cells against genotoxicity induced by alkylating treatment and oxidative stress, as already observed in other cell lines [36].

In the case of DNA double-strand break analysis, cells were treated for 1 h with 50 µM etoposide, an inhibitor of topoisomerase II that causes cytotoxic DSBs formation [39]. As the phosphorylation occurring on S139 of H2A.X, called γH2A.X, is important during DNA double-strand repair and is considered a marker of DSBs cell damage [40], we analysed the kinetics of etoposide-induced DSB repair by the different cell clones. After 1 h of etoposide treatment, cells were collected and the level of γH2A.X was measured at 0 h, 15 h and 24 h to evaluate the kinetics of repair associated with the expression of functional or non-functional APE1 protein (Fig. 1G and *Fig. S1*). As observed in Fig. 1G, the recovery from DSBs damage was strikingly different in the three cell clones tested. In the case of the APE1^NΔ33^ cell clone, the number of DSB-related γH2A.X foci remained significantly higher than controls at both release times while the amount of DSBs formation was lower in APE1^WT^ expressing clone. The repair kinetics was faster for the APE1^WT^ than control and the APE1^NΔ33^ expressing cell clones. As in the case of MMS, the expression of the NΔ33 APE1 confers a deficient phenotype to HepG2 expressing cells confirming the dominant-negative effect of this mutant toward DNA breaking lesions.

### FAs accumulation on HepG2 cells does not affect APE1 expression levels

**Figure 2:**
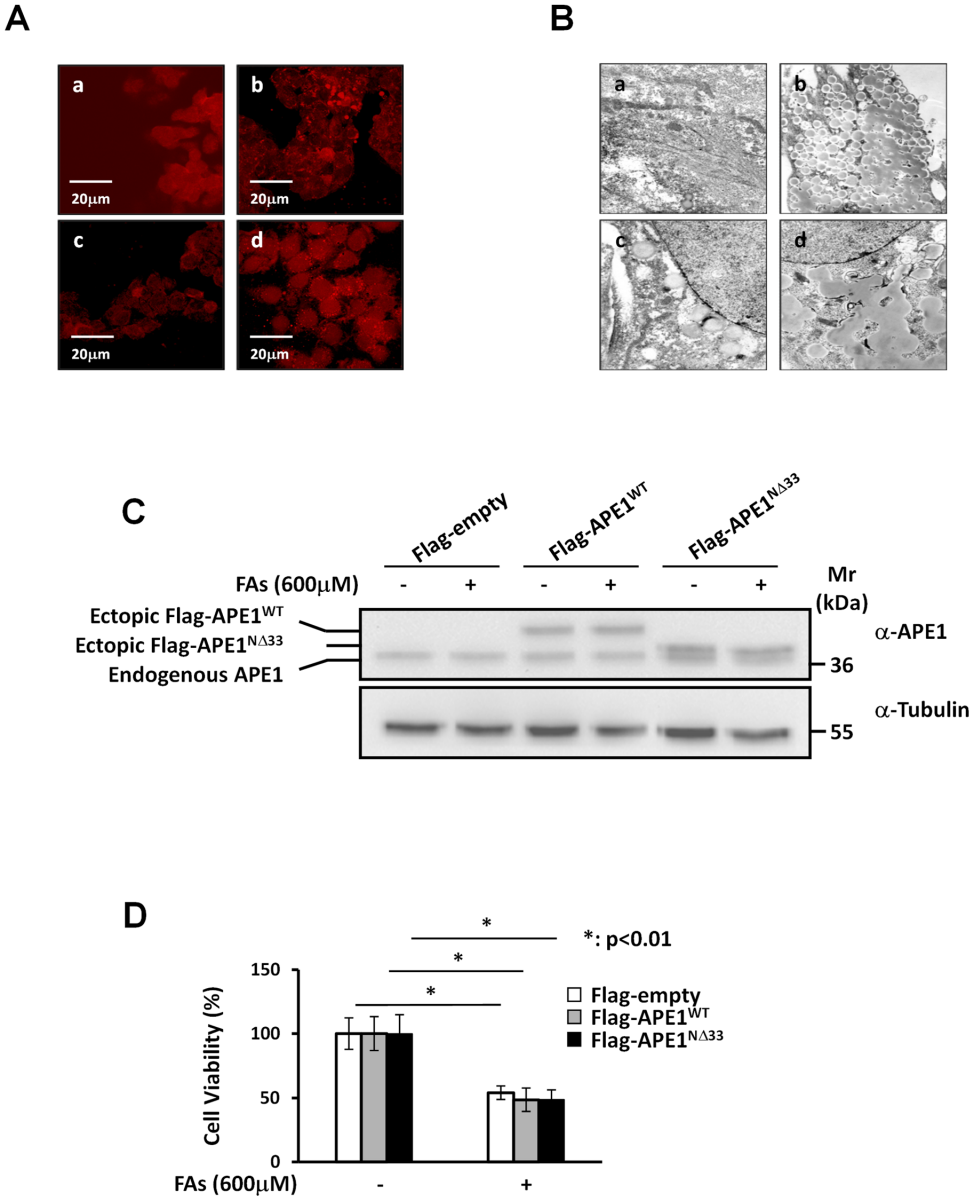
Overexpression of APE1 protein does not protect HepG2 cells from FAs treatment. **Panel A: ***Nile Red staining.***** Fluorescence of Nile Red-stained were measured on HepG2 cells previously incubated with 600 µM FAs mixture (oleate/palmitate 2∶1) for 24 h (b and d) or left untreated as control (**a** and **c**). Cells were fixed with 1.5% glutaraldehyde in PBS, washed with buffered saline and then were stained with Nile Red 10 µg/ml (**a** and **b**) or 100 μg/ml (**c** and **d**). As confirmed by Nile Red staining, HepG2 cell line exhibited a fat overloading profile. **Panel B:** ***Transmission electron microscopy analysis.*** HepG2 cells were treated with the 600 µM FAs mixture at the final ratio of 2∶1 (oleate/palmitate) for 24 h (**b** and **d**) or left untreated (**a** and **c**). Cells were then fixed and paraffin-embedded. Transmission electron microscopy confirmed fat overloading induction in HepG2 cell line. Magnification: 6300X (**a** and **b**) and 8000X (**c** and **d**). **Panel C:** ***Western Blotting analysis of total cell extracts from HepG2 stable cell clones after FAs treatment.*** HepG2 cell clones were treated with 600 µM of FAs mixture (2∶1 ratio of oleate/palmitate) for 24 h or left untreated as control. After FAs treatment, total cell extracts were prepared and 12 µg of protein extract was loaded onto a 12% SDS-PAGE, blotted and probed with anti-APE1 antibody. FAs treatment does not alter the endogenous levels of APE1 both in the APE1^WT^ and the APE1^NΔ33^ cell clones. **Panel D:** ***Effect of FAs treatment on viability of HepG2 cell clones.*** HepG2 cell clones were treated with 600 µM of FAs mixture (2∶1 ratio of oleate/palmitate) for 24 h. Cytotoxicity was assessed by trypan blue exclusion test. After exposure to FAs there was a significant reduction in cell viability but no significant difference between the clones. Data, expressed as the percentage of cell viability, are the means ± SD of three independent experiments.

To evaluate the effect of APE1 on lipotoxicity induced by FAs accumulation, HepG2 cells were incubated with 600 µM of the long-chain FAs mixture (oleate/palmitate) at the final ratio of 2∶1 for 24 h. Fat-overloading and the extent of lipid uptake, were assessed by transmission electron microscopy or by the fluorescent lipophilic dye, Nile Red, a vital staining compound used for the detection of intracellular lipid droplets by fluorescence microscopy [29] (Fig. 2A,B). As determined by Western blot analysis, the FAs treatment does not significantly alter the expression level of endogenous APE1 protein both in APE1^WT^ and APE1^NΔ33^ cell clones (Fig. 2C). Lipotoxicity also did not differ between all cell lines (Fig. 2D). These data demonstrate that, as opposed to what was observed using genotoxic treatments, overexpression of functionally active APE1 protein does not protect hepatic cells from FAs-induced cytotoxicity.

### NF-κB transcription factor regulates IL-8 promoter activity in JHH6 cells and E3330 treatment inhibits TNF-α-induced IL-8 promoter activity

**Figure 3:**
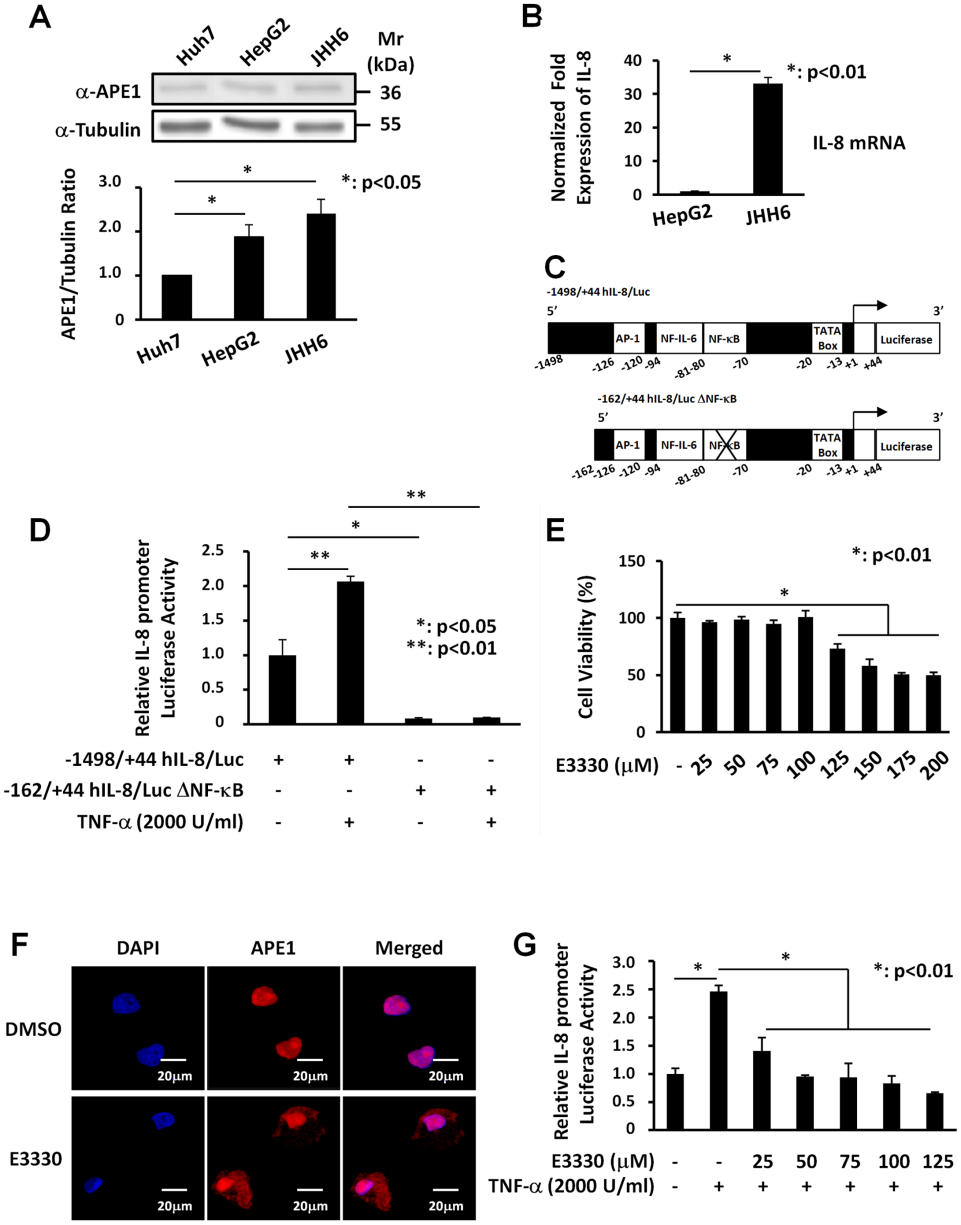
NF-κB transcription factor regulates IL-8 promoter activity in JHH6 cells and E3330 treatment inhibits TNF-α-induced promoter activation. **Panel A: ***Western Blotting analysis of total cell extracts from human hepatocellular carcinoma cell lines.***** A representative Western blot analysis for the evaluation of APE1 expression in Huh-7, HepG2 and JHH6 cell lines is shown in the upper panel. β-Tubulin was always measured as loading control and was used for data normalization. The lower panel shows expression levels of the protein obtained after densitometric analysis of the bands. An almost two-fold increase was observed in the content of APE1/Ref-1 in JHH6 cell line compared to Huh-7. Values were reported as histograms of the ratio between APE/Ref-1 band intensities and β-Tubulin. Data are the means ± SD of three independent experiments. **Panel B:** ***IL-8 mRNA expression in human hepatocellular carcinoma cell lines.*** IL-8 mRNA levels were evaluated on HepG2 and JHH6 cell lines by Real-Time PCR. Total RNA was extracted and reverse-transcribed as described in Material and Methods section. The histograms show the detected levels of IL-8 mRNA normalized to two different housekeeping genes (18S and GAPDH). An almost thirty-fold increase was observed for the mRNA IL-8 expression in JHH6 cell line. Data are the means ± SD of three independent experiments. **Panel C:** ***Schematic representation of the luciferase-linked human IL-8 promoter constructs used in this study.*** The plasmids −1498/+44 hIL-8/Luc and −162/+44 hIL-8/Luc (deleted of a 5′ promoter region) contain binding sites for AP-1, NF-IL-6 and NF-κB transcription factors. Site-directed mutation of the IL-8 NF-κB binding site in the context of the −162/+44 hIL-8/Luc plasmid abolished the binding of NF-κB on IL-8 promoter. **Panel D:** ***Effect of site-directed mutagenesis of the NF-κB binding site in the human IL-8 promoter sequence.*** JHH6 cells transfected with −1498/+44 hIL-8/Luc or −162/+44 hIL-8/Luc ΔNF-κB constructs and then treated with 2000 U/ml of TNF-α for 3 h, were analyzed through gene reporter assay. In cells transfected with the −1498/+44 hIL-8/Luc construct, TNF-α stimulated IL-8 luciferase activity, whereas mutation of the NF-κB binding site significantly decreased both basal and TNF-α-induced IL-8 promoter driven activity in JHH6. Data reported are the means ± SD of three independent experiments. These data suggest a central role of NF-κB in IL-8 gene transcription. **Panel E:** ***Effect of E3330 treatment on JHH6 viability.*** Levels of viability were measured with MTS assay in JHH6 cells treated for 7 h with increasing doses of E3330. Up to a concentration of 100 µM the treatment with E3330 did not affect the cellular viability. Data, expressed as the percentage of cell viability, are the means ± SD of three independent experiments. **Panel F:** ***Effect of E3330 on APE1 subcellular distribution.*** APE1 subcellular localization was detected through confocal microscopy analysis using a specific α-APE1 monoclonal primary antibody. APE1 mainly localized within the nuclear compartment and accumulated into nucleoli. Treatment with 100 µM E3330 for 6 h induced a robust cytoplasmic enrichment of APE1. As control, cells were treated with DMSO without any effect on APE1 subcellular distribution. **Panel G:** ***Effect of E3330 treatment on TNF-α-induced IL-8 promoter activity.*** JHH6 cells transfected with −1498/+44 hIL-8/Luc construct were pre-treated with increasing concentration of E3330, or with vehicle (DMSO) as a control, for 4 h prior to treatment with 2000 U/ml TNF-α for 3 h. TNF-α stimulated IL-8 luciferase activity and the pre-treatment with E3330 significantly decreased, in a dose-dependent manner, TNF-α-induced IL-8 promoter activity. Data reported are the means ± SD of three independent experiments.

IL-8 gene expression is mainly modulated at the transcriptional level in a stimulus- and cell type-specific manner [41]. High levels of IL-8 mRNA and APE1 protein expression may play an important role in inflammatory liver injury as a consequence of lipid accumulation. To determine the proper experimental model in which testing the causal link between APE1 and IL-8 expression, we initially determined the expression levels of these two genes in Huh-7, HepG2 and JHH6cell lines . As shown in figure 3A, protein expression was highest in JHH6 cells, which express approximately 2.5-fold higher levels of APE1 protein than control Huh-7 cells. Then, we compared the expression levels of IL8 gene in HepG2 and JHH6 cells through RT-PCR (Fig. 3B) and observed that JHH6 cells express higher levels of IL8 than HepG2 cells. These observations indicate that, with respect to APE1 expression levels, JHH6 cells are a good model to investigate the role of APE1 in the inflammatory process associated to liver tumorigenesis.

IL-8 transcriptional responses to pro-inflammatory mediators are rapid and require only 100 nucleotides of 5′-flanking DNA upstream of TATA-box. Within the IL-8 promoter sequence, DNA binding sites for inducible transcription factors AP-1, NF-IL-6 and NF-κB are present [30], [42]. To investigate the role of NF-κB on basal and TNF-α-induced IL-8 expression in JHH6 cells, we evaluated the effect of deletion of the NF-κB binding site on IL-8 promoter sequence. We used a reporter assay approach to study the effect of TNF-α on the IL-8 expression levels. Cells were transiently transfected with luciferase-linked constructs of the wild-type human IL-8 promoter −1498/+44 hIL-8/Luc and the −162/+44 hIL-8/Luc ΔNF-κB in which the NF-κB binding sites were deleted [30] (Fig. 3C). After transfection cells were stimulated with TNF-α for 3 h to trigger NF-κB functional activation. As shown in Fig 3D, TNF-α treatment significantly stimulated the IL-8-promoter activity only in cells transfected with the −1498/+44 hIL-8/Luc plasmid while TNF-α stimulatory effect was completely lost in cells transfected with deletion mutant promoter. Notably, the basal transcriptional activity of the IL-8 promoter was dependent on the presence of functional NF-κB binding sites suggesting that the transcription factor NF-κB plays a central role in both basal and TNF-α-induced IL-8 promoter activity.

Given that APE1 regulates NF-κB DNA binding activity in a redox-dependent manner [11], we speculated if the specific inhibitor of APE1 redox function, E3330 [16], was able to reduce TNF-α-induced IL-8 gene expression. Accordingly, we evaluated the effect of E3330 on cell viability. Analysis using the MTS assay on JHH6 cells treated for 7 h with several doses (25–200 µM) of E3330 revealed that E3330 has no cytotoxic effect up to a concentration of 100 µM (Fig. 3E). We determined whether E3330 treatment impairs subcellular APE1 trafficking in hepatic cells, as previously observed in glioblastoma cells [17]. Immunofluorescence experiments (Fig. 3F) clearly demonstrated that a significant relocalization of APE1 protein from the nuclear to the cytoplasmic compartment occurred in JHH6 cells with no significant alterations in total protein levels, as measured by Western blot analysis (data not shown). We then examined the effect of E3330 on TNF-α-induced IL-8 promoter activation. JHH6 cells were pre-treated with increasing concentrations of E3330, for 4 h prior to treatment with TNF-α for 3 h. As shown in figure 3G, pre-treatment with E3330 significantly attenuated TNF-α-induced IL-8 promoter activity in a dose-dependent manner.

### E3330 treatment specifically decreases both TNF-α- and FAs-induced IL-8 endogenous gene expression

**Figure 4:**
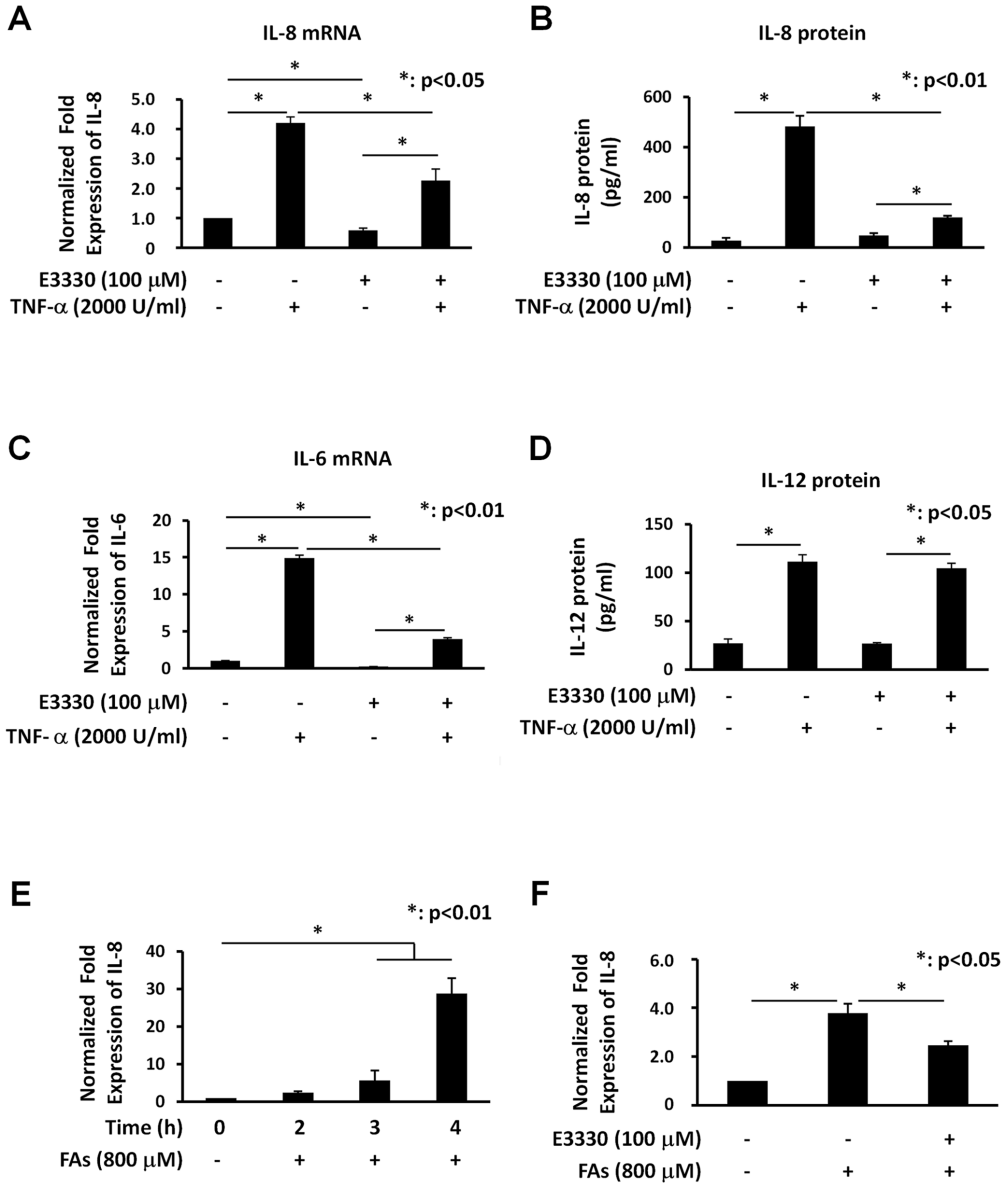
E3330 treatment specifically inhibits TNF-α- and FAs-induced IL-8 endogenous gene expression. **Panel A: ***Effect of E3330 treatment on TNF-α-induced IL-8 mRNA expression.***** JHH6 cells were pre-treated with 100 µM E3330 or with vehicle (DMSO) as a control, for 4 h prior to treatment with 2000 U/ml TNF-α for 2 h. IL-8 mRNA expression was determined by Real-Time PCR. The histograms show the detected levels of IL-8 mRNA normalized to control (DMSO) and normalized to two different housekeeping genes (GAPDH and HPRT). IL-8 mRNA expression was increased by TNF-α treatment when compared with control cells and pre-treatment with 100 µM E3330 decreased TNF-α-induced IL-8 mRNA. Data reported are the means ± SD of three independent experiments. **Panel B:** ***Effect of E3330 treatment on TNF-α-induced IL-8 protein production.*** JHH6 cells were pre-treated with 100 µM E3330 or with vehicle (DMSO) as control, for 4 h prior to treatment with 2000 U/ml TNF-α for 2 h. The supernatants of the same cells analyzed for mRNA were assayed for IL-8 protein by FlowCytomix assay kit. TNF-α stimulated the secretion of IL-8 protein by JHH6 cells and the pre-treatment with 100 µM E3330 significantly suppressed TNF-α-induced IL-8 protein release. Data reported are the means ± SD of three independent experiments. **Panel C:** ***Effect of E3330 treatment on TNF-α-induced IL-6 mRNA expression in JHH6 cells.*** JHH6 cells were pre-treated with 100 µM E3330 or with vehicle (DMSO) as a control, for 4 h prior to treatment with 2000 U/ml TNF-α for 3 h. IL-6 mRNA expression was determined by Real-Time PCR. The histograms show the detected levels of IL-6 mRNA normalized to control (DMSO) and normalized to two different housekeeping genes (GAPDH and HPRT). IL-6 mRNA expression was increased by TNF-α treatment and the pre-treatment with 100 µM E3330 significantly decreased TNF-α-induced IL-6 mRNA. Data reported are the means ± SD of three independent experiments. **Panel D:** ***Effect of E3330 treatment on TNF-α-induced IL-12 protein production in JHH6 cells.*** The same supernatants analyzed for IL-8 protein were assayed for IL-12 protein by FlowCytomix assay kit. E3330 does not affect TNF-α-induced IL-12 activation suggesting a specific effect of E3330 on IL-8 gene expression. Data reported are the means ± SD of three independent experiments. **Panel E:** ***Effect of FAs overload on IL-8 gene expression.*** JHH6 cells were treated for different times with 800 µM of mixture of oleate/palmitate (2∶1 ratio) and IL-8 mRNA expression was determined by Real-Time PCR. The histograms show the detected levels of IL-8 mRNA normalized to control (DMSO) and normalized to two different housekeeping genes (GAPDH and HPRT). IL-8 mRNA expression was increased by FAs treatment when compared with control cells in a time-dependent manner. Data reported are the means ± SD of three independent experiments. **Panel F:** ***Effect of E3330 treatment on FAs-induced IL-8 mRNA expression.*** JHH6 cells were pre-treated with 100 µM E3330 or with vehicle (DMSO) as a control, for 4 h prior to treatment with 800 µM of mixture of oleate/palmitate (2∶1 ratio) for 3 h. IL-8 mRNA expression was determined by Real-Time PCR. The histograms show the detected levels of IL-8 mRNA normalized to control (DMSO) and normalized to two different housekeeping genes (GAPDH and HPRT). IL-8 mRNA expression was increased by FAs treatment and pre-treatment with 100 µM E3330 decreased FAs-induced IL-8 mRNA. Data reported are the means ± SD of three independent experiments.

To investigate the effect of E3330 on TNF-α-induced IL-8 expression, JHH6 cells were pre-treated with 100 µM E3330 for 4 h before exposure to TNF-α. As shown in figure 4, pre-treatment with E3330 significantly decreased TNF-α-induced IL-8 expression in terms of both endogenous mRNA transcription (Fig. 4A) and protein production (Fig. 4B). We confirmed the inhibitory effect of E3330 on IL-6 expression (Fig. 4C), another cytokine activated by NF-κB which is induced by FAs accumulation. Interestingly, E3330 does not affect TNF-α-induced IL-12 protein secretion suggesting a specific effect of E3330 on IL-8 and IL-6 gene expression, at least in our experimental system (Fig. 4D). These data point to a potential use of E3330 to reduce the inflammatory processes in liver diseases such as those associated with NASH.

To investigate whether FAs overloading could play a role in the development of inflammation through induction of IL-8 production, we used the *in vitro* cell model of hepatic steatosis, i.e. JHH6 cells, described above. JHH6 cells were treated for different times with 800 µM of mixture of oleate/palmitate (2∶1 ratio). This treatment had not effect on cell viability (data not shown) while IL-8 mRNA expression was increased in a time-dependent manner (Fig. 4E). To investigate the effect of E3330 on FAs-induced IL-8 expression, JHH6 cells were pre-treated with 100 µM E3330, for 4 h prior to treatment with 800 µM FAs for 3 h. As shown in Figure 4F, pre-treatment with E3330 significantly decreased FAs-induced IL-8 expression.

## Discussion

The current work was aimed at elucidating the role and the impact of APE1 in the onset of inflammatory circuits in liver diseases, such as those associated to NASH and to evaluate whether the APE1 redox inhibitor, i.e. E3330, may prevent the induction of IL-8 and IL-6 expression by both TNF-α or FA treatment.

**Figure 5:**
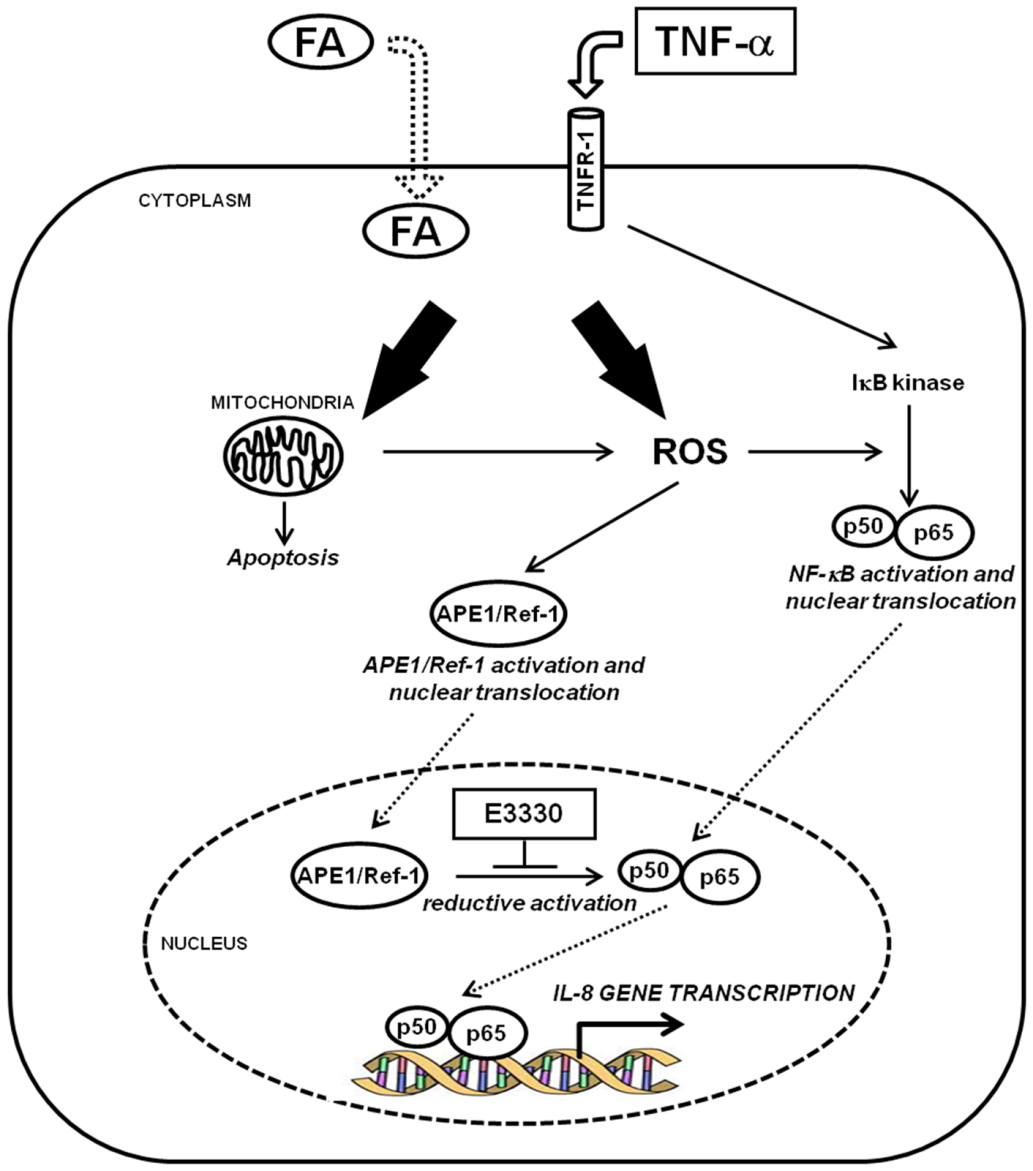
Model of the effect of E3330 redox inhibitor on the Fatty Acid-TNFα-APE1-NFκB-IL8 axis. APE1 redox inhibitor E3330 prevents inflammatory cytokines production (IL-8 and IL-6) triggered by FAs accumulation and TNF-α stimulation in hepatic cancer cell lines. In this pathway, mitochondrial impairment and resulting oxidative stress condition may cause the functional activation of NF-κB transcription factor through APE1 regulatory redox function leading to IL-8 and IL-6 gene expression.

Our data demonstrate that in hepatic cancer cell lines APE1 redox function is involved in TNF-α and FA-induced IL-8- and IL-6 expression, and its inhibition by E3330 may represent a promising tool for reducing the early inflammatory process in liver diseases such as in NASH. NASH is a clinically relevant pathology since the high prevalence in the general population and the possible evolution towards pathologies with a fatal outcome [2]. NASH has been correlated with direct lipid toxicity, impaired mitochondrial function, elevated cytochrome P450 activity (specifically CYP2E1 and CYP4A10/4A14), oxidative damage and increased inflammatory cytokines levels in the liver and periphery [9]. Since a better understanding of the molecular events regulating these mechanisms may be helpful in designing new therapeutic strategies, we utilized an *in vitro* hepatic model to study the pathway responsible for inflammatory cytokines production (IL-8 and IL-6) triggered by FAs accumulation and TNF-α stimulation (Fig. 5). In this pathway, mitochondrial impairment and resulting oxidative stress condition may cause the functional activation of NF-κB transcription factors through APE1 regulatory redox function leading to IL-8 and IL-6 gene expression.

To create a lipotoxic condition and a pro-inflammatory status, we treated JHH6 cells with FAs or TNF-α. In fact, it has been demonstrated in different models that FAs increase the expression of inflammatory cytokine through mechanism involving activation of NF-κB [7], [8], [9]. Also, TNF-α plays an important role in inflammatory liver injury. Accordingly, intrahepatic gene expression and/or plasma levels of TNF-α are increased in fatty liver and in NASH patients [43] and modulation of TNF-α expression by genetic deletion or other means results in the amelioration of steatosis, inflammation, and hepatocyte damage in *ob/ob* mice and in dietary models of steatohepatitis [44] suggesting a pivotal role of this cytokine in NASH. Moreover, interference with NF-κB activation significantly protected from the development of steatohepatitis, and reduced the expression of TNF-α and ICAM-1 [45]. These observations suggest that TNF-α is one of the multiple effectors of steatohepatitis, and that NF-κB activation plays a pivotal role in the early onset and maintenance of the inflammatory process.

In the molecular pathway represented in Figure 5, the cellular damage due to FAs or TNF-α treatment is principally mediated by mitochondrial impairment. In fact the impairment in free FAs β-oxidation is at the basis of the onset of NASH. Mitochondria are involved in both free FA β-oxidation and reactive oxygen species (ROS) generation. Patients with NASH are characterized by abnormal mitochondria from both a functional and a morphological point of view. Accumulating evidence indicates that respiratory chain defects are key-determinant of mitochondrial dysfunction and thus oxidative stress generation [9]. Chemical modification of essential biomolecules by ROS and RNS cause their functional inactivation and lead to either cell death or to an adaptive cellular response, e.g. activation of redox-sensitive transcription factors [46] (nuclear factor NF-κB, Nrf-1 and Sp-1) contributing to the production of pro-inflammatory and fibrogenic mediators by Kupffer cells and HSC. Of interest is the recent finding of a role for the transcription factor Nrf-1, a central player in controlling expression of antioxidant genes through binding to ARE sequences, in NASH and hepatic cancer development [47]. Interestingly, both Nrf-1 and APE1 are over-expressed in liver cancer [14]. Very recently, Li *et al* demonstrated that APE1 acts as a new redox coactivator of Nrf-1 regulating the expression of *Tfam*, *Cox6c*, and *Tomm22* nuclear genes with mitochondrial function. This evidence highlights an additional regulatory role for APE1 in the maintenance of mitochondrial functionality upon oxidative stress and provides the first hypothesis for an indirect mitochondrial function of APE1 through a nuclear transcriptional mechanism [48]. Collectively, these findings suggest that the APE1-Nrf-1 axis deserves further attention.

For a better understanding of the role of APE1 in the onset of inflammatory circuits in liver diseases, we wondered whether APE1 overexpression would protect hepatic cells from the cytotoxic effects of genotoxicants and from lipid induced cytotoxicity. We used HCC-derived cell line overexpressing functional and non-functional APE1 proteins. Due to the prominent role played by APE1 in the repair of specific oxidative, alkylation, and enzymatic DNA intermediates, we treated cells with different DNA damaging agents, including MMS, hydrogen peroxide and etoposide. The results show the expected protective function of APE1 towards these genotoxic agents [36], [49], confirming the validity of the cell model developed. Moreover, they suggest that the upregulation of APE1 protein expression levels observed in hepatic cancer cells may reflect an adaptive response to cell damage and may be responsible for further cell resistance to chemotherapy, as shown in other cancer cell models [49], [50]. However, the same protection was not evident in the case of FAs cytotoxicity. Data obtained also suggested that the increase of APE1 protein observed both in NASH models and in liver tissue obtained from NASH patients [13] may result as the consequence of lipid-induced hepatocellular injury. The damage may be explained on the basis of APE1′s roles in intracellular signalling as a transcriptional coactivator rather than its role as DNA repair enzyme. The deficient phenotype, observed in the case of the NΔ33 mutant expressing cells, confirms the dominant-negative effect of this mutant toward DNA breaking lesions. This effect may be due to the loss of BER coordination as the consequence of the impairment of APE1 protein-protein interactions (which is in fact modulated by the first 33–35 N-terminal amino acids of APE1) and to the concurrent competition of the NΔ33 mutant with the WT form for the same damaged substrates [17], [33].

Since APE1 is upregulated in hepatic cancer cells, the APE1 overexpression cell model used for this study can be compared to the condition observed in advanced stages of liver cancer progression [14]. The protective role of APE1 from genotoxicants suggest that its up-regulation may occur as an adaptive response to cell damage and may be associated with the onset of cancer resistance. Since APE1 has emerged as an excellent target for sensitizing tumor cells to chemotherapy [50], [51], [52], [53], APE1 DNA-repair and redox-inhibition may be used as a promising strategy for liver cancer treatment [34], [54]. Recent studies have demonstrated a direct effect of E3330 on a variety of cancer cells [55], [56] and that E3330 has potential efficacy in pancreatic cancer. In fact, inhibition of APE1 via E3330 results in tumor growth inhibition in cell lines and pancreatic cancer xenograft models in mice. These effects of E3330 are accomplished through the redox inhibition of APE1 on the activity of NF-κB, AP-1, and HIF1α that are key transcriptional regulators involved in survival and invasion, and may lead to the blockage of the metastatic process [20]. E3330 directly interact with APE1 and inhibits its redox activity by increasing the formation of disulfide bonds by Cys-65, increased unfolding of the protein, and decreasing the protein redox active population [15]. APE1 subcellular distribution within different mammalian cell types is mainly nuclear and critically controls cellular proliferative rate [37], [57]. We recently demonstrated, in the human glioblastoma cell line SF767, that E3330 treatment caused a significant relocalization of APE1 from the nuclear to the cytoplasmic compartment with no significant alterations in total protein levels. Moreover, kinetic experiments also demonstrated that E3330 treatment caused a progressive emptying of the nucleoli. These data demonstrate that some of E3330 effects are associated with alterations of APE1 trafficking and that the redox state of C65, or the altered configuration of the APE1 protein, may control APE1 cellular distribution providing the basis for a new role for this residue in controlling subcellular distribution [17]. In this study, we demonstrated that in JHH6 hepatic cancer cells, E3330 treatment inhibits TNF-α-induced IL8 production through impairment of APE1 subcellular distribution, thus confirming a novel aspect of E3330 effect on APE1 modulation. It would also be interesting to check whether the redox state of APE1 may explain the cytoplasmic accumulation already observed in HCC [14].

In comparison with other cell types as pancreatic cell lines [58] we noticed that the hepatic cancer cell lines were less sensitive to E3330 treatment in terms of cell viability. For example, in PDAC pancreatic cancer cell line and primary patient cells, E3330 has little killing or growth effect until concentrations greater than 67.5 µM are reached [20], [59]. Other studies using E3330 at concentrations similar to or above 100 µM have been published including cellular EMSA assays [60], a reduction of APE1 nuclear location after 140 µM E3330 treatment without cell killing [17], and cell survival in human hepatocellular cell lines [61] after 100 µM treatments. Recent data obtained in LPS-activated macrophages, demonstrated that E3330 suppresses the inflammatory response. *Via* the inhibition of APE1 redox function on NF-κB and AP-1 transcriptional activity, E3330 suppresses secretion of inflammatory cytokines including TNF-α, IL-6 and inflammatory mediators such as nitric oxide and prostaglandin E(2) [21]. These results and data presented here are in agreement with other reports demonstrating the hepatoprotective effect of E3330 against endotoxin-mediated hepatitis and alcoholic liver injury. In fact in endotoxin-induced murine hepatitis models, E3330 attenuates the elevation of plasma TNF activity suggesting that E3330 protects mice from liver injury through the inhibition of TNF production [62]. In an experimental alcoholic liver injury rat model, E3330 reduces thromboxane B2 and leukotriene B4 levels in both nonparenchymal cell supernatant and plasma and reduces TNF levels in nonparenchymal cell supernatant. These findings suggest that E3330 has a protective effect in alcoholic liver injury trough inhibition of thromboxane, leukotriene and TNF generation [63]. All these observations underscore the relevance of using E3330 for treatment of inflammatory-based liver diseases and the need of further in vivo studies in NASH animal models.

## Supporting Information

Figure S1 **Immunofluorescence staining for double strand DNA damage.** HepG2 cell clones were incubated with or without etoposide (50 µM) for 1 h. After the incubation and the time course for the release, cells were fixed, permeabilized and stained for the phosphorylated form of the histone H2A.X using a specific antibody (green). Nuclei were detected with Propidium Iodide (red). Scale bar correspond to 20 µm.
(TIF)
