# Correction: Specific Inhibition of the Redox Activity of Ape1/Ref-1 by E3330 Blocks Tnf-Α-Induced Activation of Il-8 Production in Liver Cancer Cell Lines

**DOI:** 10.1371/annotation/a610ee5c-525c-4d3c-b6b7-a4cde7b8db54

**Published:** 2013-08-22

**Authors:** Laura Cesaratto, Erika Codarin, Carlo Vascotto, Antonio Leonardi, Mark R. Kelley, Claudio Tiribelli, Gianluca Tell

There was an error in the title.

The correct version of the title is: Specific Inhibition of the Redox Activity of Ape1/Ref-1 by E3330 Blocks Tnf-α-Induced Activation of Il-8 Production in Liver Cancer Cell Lines

The correct citation is: Cesaratto L, Codarin E, Vascotto C, Leonardi A, Kelley MR, et al. (2013) Specific Inhibition of the Redox Activity of Ape1/Ref-1 by E3330 Blocks Tnf-α-Induced Activation of Il-8 Production in Liver Cancer Cell Lines. PLoS ONE 8(8): e70909. doi:10.1371/journal.pone.0070909 

