# Supplementary figures and images for: Specific Inhibition of the Redox Activity of Ape1/Ref-1 by E3330 Blocks Tnf-Α-Induced Activation of Il-8 Production in Liver Cancer Cell Lines
Source: PLoS One. 2013 Aug 15;8(8):e70909. doi: 10.1371/journal.pone.0070909 (PMC3744539; doi:10.1371/journal.pone.0070909)

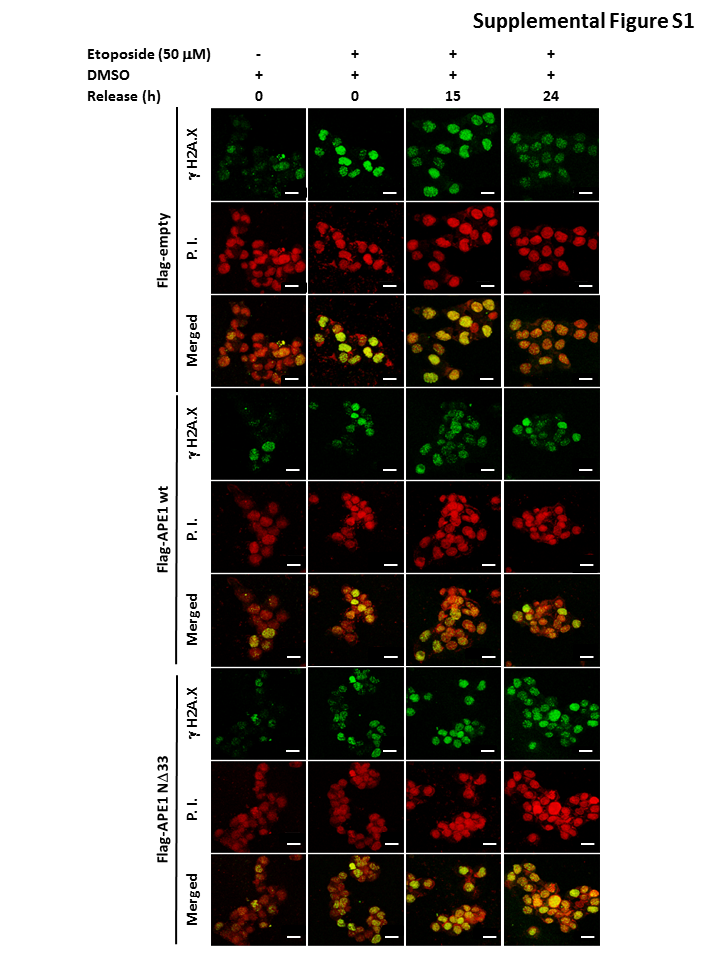

Supplement: Figure S1 — Immunofluorescence staining for double strand DNA damage. HepG2 cell clones were incubated with or without etoposide (50 µM) for 1 h. After the incubation and the time course for the release, cells were fixed, permeabilized and stained for the phosphorylated form of the histone H2A.X using a specific antibody (green). Nuclei were detected with Propidium Iodide (red). Scale bar correspond to 20 µm. (TIF) [file pone.0070909.s001.tif]
